# Pedicled Soleus Muscle Flap for Salvage Therapy of Chronic Limb-Threatening Tibial Osteomyelitis: A Case Report

**DOI:** 10.7759/cureus.22086

**Published:** 2022-02-10

**Authors:** Brett J Matoian, Robert J Dabek, George Grace

**Affiliations:** 1 General Surgery, Saint Agnes Hospital, Baltimore, USA; 2 Plastic and Reconstructive Surgery, Saint Agnes Hospital, Baltimore, USA

**Keywords:** limb salvage surgery, osteomyelitis, opiod epidemic, intravenous drug abusers (ivda), limb reconstruction

## Abstract

The resurgence of opiate and intravenous drugs abuse in the United States has presented a renewed challenge to surgeons in community-based hospital settings. Patients often present with complex wounds, and when complicated by concomitant osteomyelitis, these wounds require special attention and diligent care. Local rotational flaps have been used in the salvage therapy of limb-threatening lower extremity trauma for years, and have been adapted in part for the use in patients with chronic, limb-threatening osteomyelitis. The use of local rotational flaps for coverage of chronic osteomyelitis is a viable, proven, and well-founded surgical technique with excellent results. Within our hospital setting, we have seen an explosion of patients presenting with chronic, limb-threatening wounds related to intravenous and subcutaneous injection of a variety of illicit drugs.

Here, we describe a case of a 24-year-old female with a history of intravenous drug abuse (IVDA) who presented with an extensive left lower extremity wound which had been progressing for several years. The patient was acutely intoxicated but otherwise healthy. Due to extensive tissue loss and osteomyelitis, initial evaluation deemed her leg unsalvageable. However, given the immense morbidity associated with lower extremity amputation the plastic surgery team felt that salvage should be attempted in this young woman. She underwent numerous tissue debridements, washouts, cadaveric skin grafting, and a pedicled soleus muscle flap with eventual autologous skin grafting. The patient was kept in the hospital during this time to allow her to detox and undergo psychiatric evaluation and therapy. This approach allowed her to regain nearly full use of her limb, gain employment, as well as abstain from further drug use.

As the opioid epidemic continues in inner cities throughout the United States, the increased burden on local medical centers to care for chronic limb-threatening wounds will continue to rise. Locoregional flaps provide good results but may not be suitable for unreliable patients struggling with addiction. However, in motivated patients, our approach of inpatient detox and delayed reconstruction has shown promising results.

## Introduction

Plastic and reconstructive surgeons attempting salvage therapy of the lower extremity are often faced with the challenge of limited local soft tissue options for coverage. Free flaps have been used effectively for decades, however, they are faced with high costs in terms of surgical expertise, length of operation, and specific systems required for postoperative care [1,2]. Over the past 10-15 years, with increased knowledge of lower extremity vascular anatomy and the advent of the wound vacuum-assisted closure (VAC) device, there has been a trend toward increased proportional use of local rotational flaps over free microvascular flaps for coverage of the middle and distal third lower leg [3]. When used effectively, soleus flaps have been found to be reliable, provide a cosmetically appropriate outcome, and are relatively cost- and resource-efficient [3].

Though most often documented in the reconstruction of lower extremity trauma, soleus and other local flaps are also used successfully for the salvage therapy of severe, limb-threatening lower extremity infection, and osteomyelitis [4]. When considering severely infected wounds, it is well-established that the use of muscle flap coverage aids in healing by protecting local debridement and improving the vascularity necessary for delivery of long-term antibiotic therapy [5].

Osteomyelitis as a disease process is increasingly problematic in the American population as risk factors for its development, including diabetes and the use of intravenous drugs continue to rise [6]. This latter category presents a unique problem for the limb salvage surgeon as the use of IV opiates contributes greatly to the development of limb-threatening infection [7]. Additionally, it is frequently found in younger populations whose subsequent loss of an extremity portends the potential for a loss of greater quality of life years [8]. 

To emphasize the importance of the soleus flap to the limb salvage operation, and to present a thought-provoking case encountered in an innercity hospital with expertise in limb salvage, we present the case of a 24-year-old female with active intravenous drug abuse (IVDA) and subsequent development of a severe chronic lower extremity ulcer associated with osteomyelitis of the left tibia and fibula. This wound was cared for and salvaged with a combination of vascular and plastic surgical teams at Ascension Saint Agnes Hospital in Baltimore, Maryland.

## Case presentation

**Figure 1:**
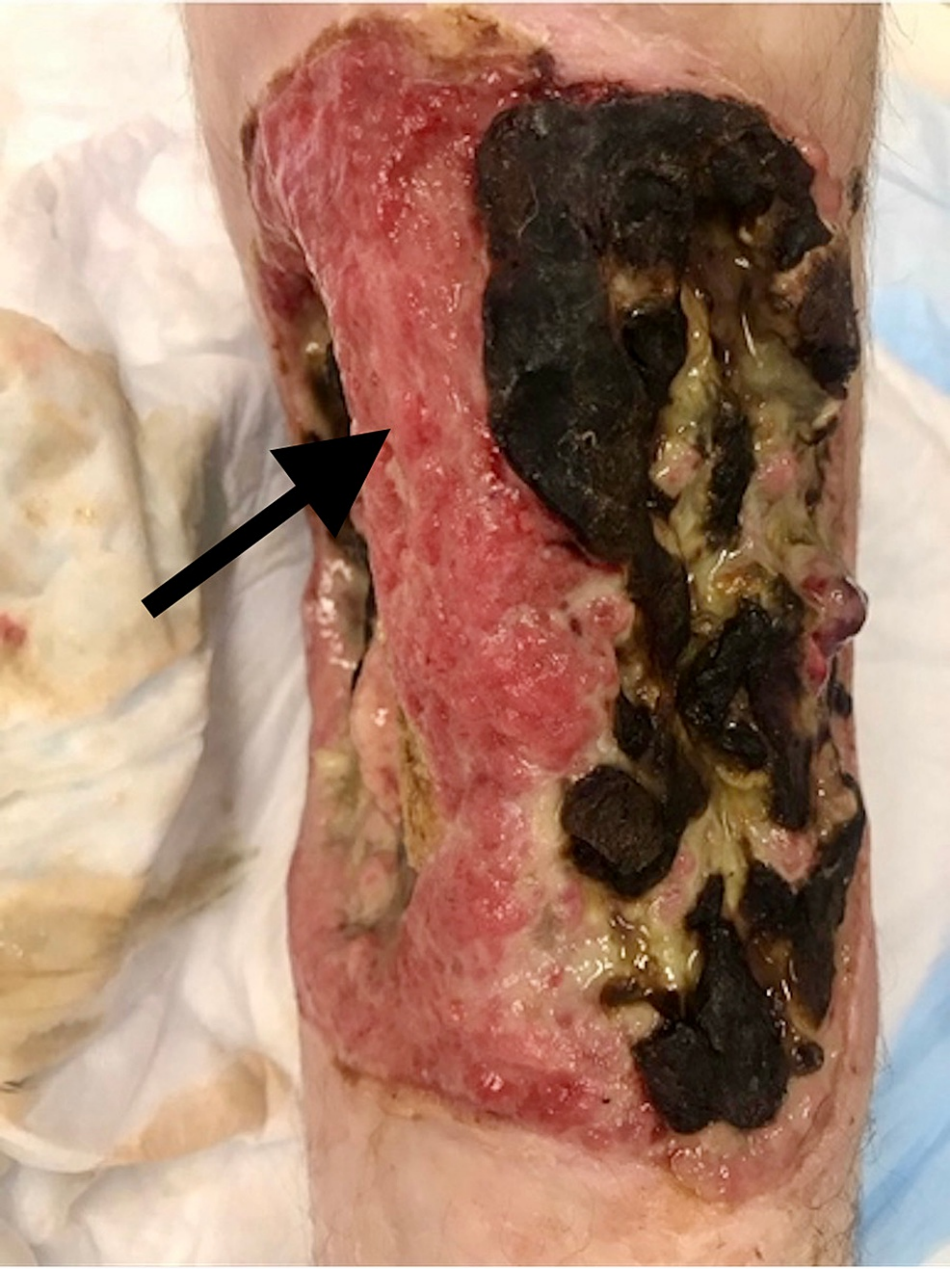
Large, near-circumferential ulcer at the level of the mid-leg with exposed bone. Black arrow shows tibia and medial leg. Superior portion of the image is just distal to knee joint and inferior portion of the image marks just superior to ankle.

**Figure 2:**
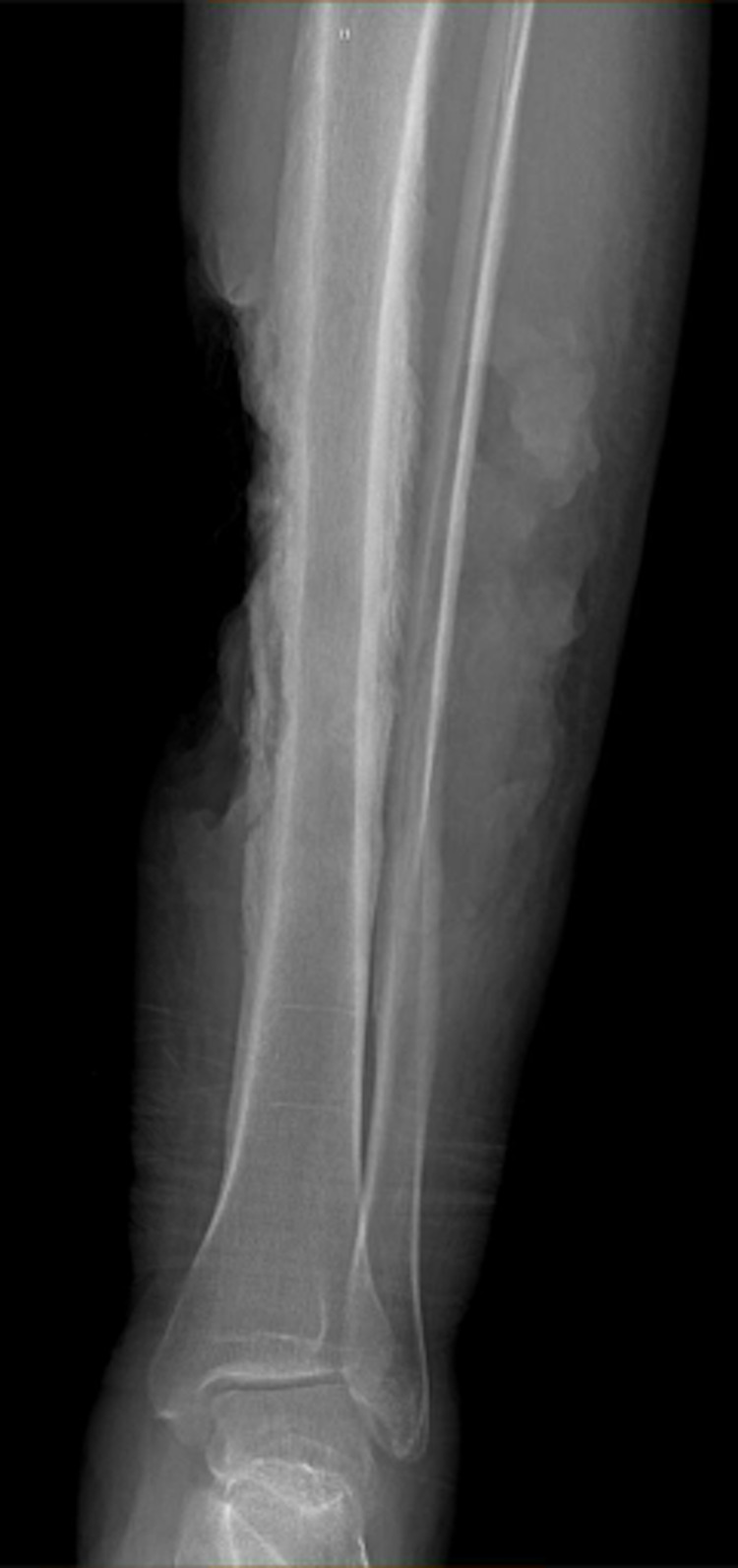
X-ray imaging reveals extensive periosteal reaction across the mid to distal tibia, which is suspicious for osteomyelitis.

A 24-year-old female with a history of IVDA presented with an extensive left lower extremity wound which had been progressing for several years. Prior to her admission, she had been seen at a community wound clinic but had stopped attending appointments several months earlier due to lack of transportation. Subsequently, the severity of the wound worsened and began to cause considerable pain and disability (Figures 1, 2). Initially, the vascular surgical service was contacted to evaluate the patient for a left below-knee amputation. Due to the extensive soft tissue involvement, muscle loss, and concern for osteomyelitis, the limb was felt to be non-salvageable. However, subsequent CTA of the extremity found adequate two-vessel runoff to the foot (absent posterior tibial) which was deemed adequate for a limb salvage attempt. In addition, the patient expressed sincere interest in recovery from her opioid dependence, which was considered an important consideration given the risk of successfully undergoing reconstruction in an active intravenous drug user.

Initially, the patient was started on broad-spectrum antibiotics and scheduled for operative intervention. During her first OR visit, she underwent aggressive mechanical and sharp debridement in addition to jet lavage. Bone and soft tissue biopsies were obtained as exposed bone was found in the wound. The wound was then covered with a VAC system and the patient was transported back to the floor for recovery. Superficial wound cultures grew mixed Gram-negative rod bacterium and final bone pathology of the fibula was positive for acute osteomyelitis with partial necrosis. With these results obtained, a process of long-term antibiotic coverage was begun via a peripherally inserted central line, and the patient began detoxification from her opiate dependence. The next series of operations occurred in three-day increments, where the patient returned to the operating theatre every three days for additional light mechanical debridement, cadaveric skin graft placement, and placement of a VAC device.

**Figure 3:**
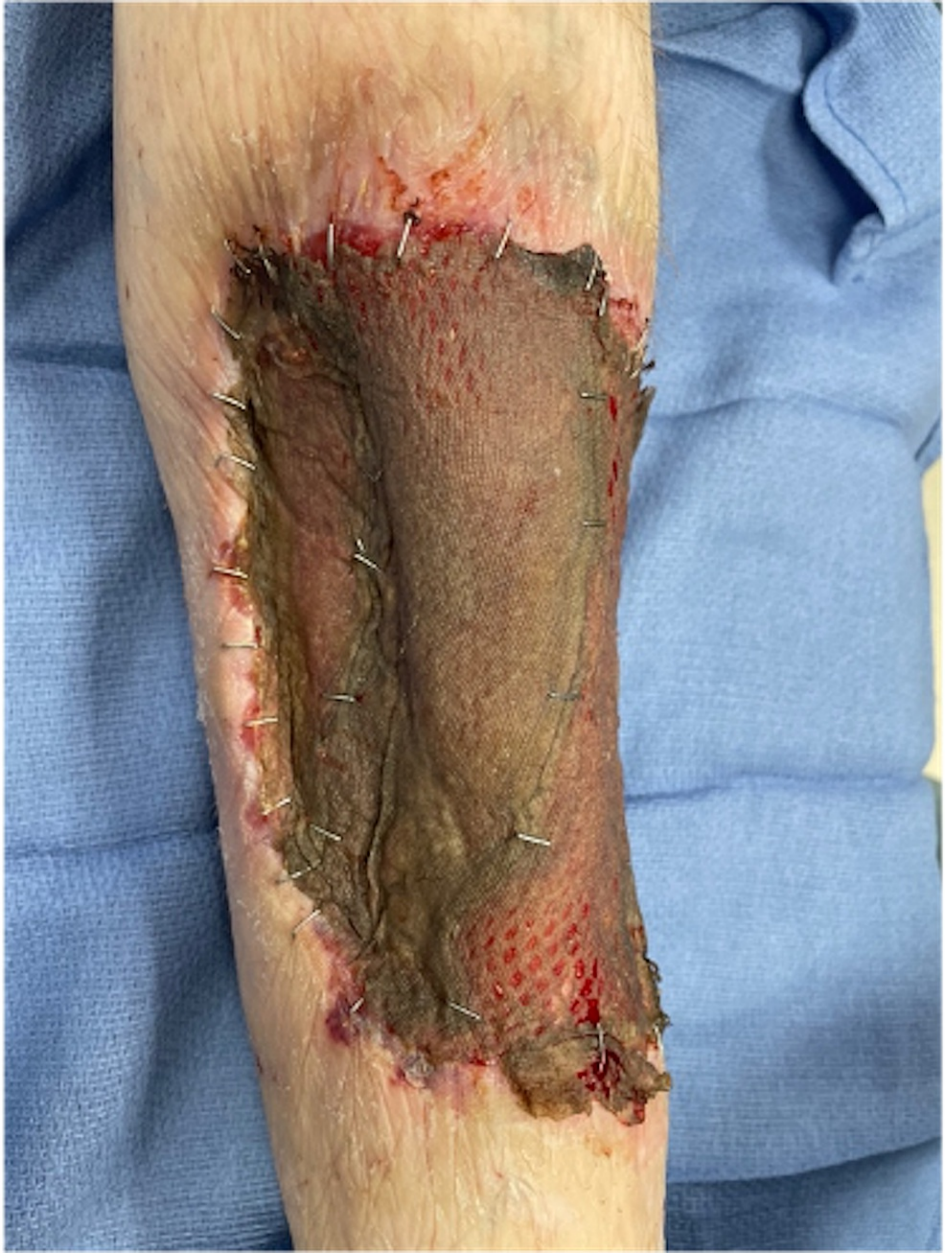
Cadaveric skin graft three days following initial aggressive soft tissue debridement. Medial portion of the leg is left side of the image, ankle oriented toward the bottom of the image.

Three days after the initial operation, she was taken back to the operative theatre for a second look, and ultimately began a process of sequential cadaveric skin graft placement (Figure 3). The wound was covered with a negative pressure VAC system, and the patient was returned to the floor for recovery. During this procedure, it was noted that the only area which demonstrated inadequate granulation tissue formation was overlying portions of the wound in which there was exposed bone. This highlighted the need for possible soft tissue coverage to adequately clear the infection and heal that portion of the wound.

During the patients' third operation, the skin graft was removed, the wound underwent gentle mechanical debridement, which demonstrated healthy bleeding granulation tissue and the extremity was jet lavaged. No additional debridement was found to be necessary, an additional cadaveric skin graft was applied in the defect, meshed to 1:1.5, and a VAC dressing was applied.

**Figure 4:**
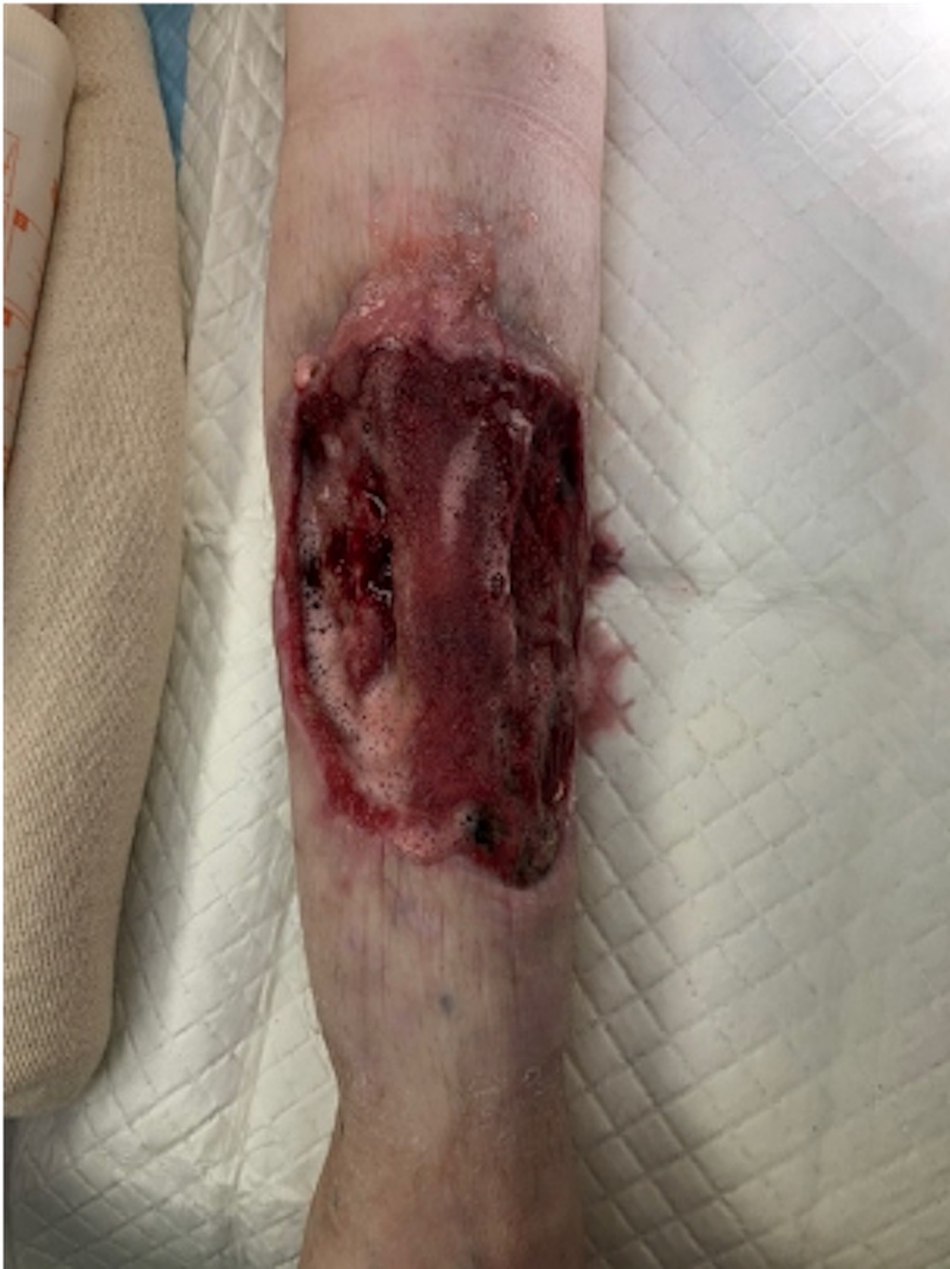
Image showing the evidence of healthy granulation tissue formation, following removal of cadaveric skin graft. Ankle is oriented toward the bottom of the image. Exposed tibia is noted in the middle of the wound. Medial aspect of the leg is oriented toward the left side of the image.

Three days later, she returned to the operating room for fourth time. A bone burr was used to smooth the area of the bone biopsy, which produced excellent punctate bleeding, the cadaveric skin was removed, the wound was jet lavaged, and few remaining areas of chronic old granulation tissue were sharply debrided (Figure 4).

The rotational soleus flap was created using a lower curvilinear incision over the posterior aspect of the leg. Due to chronic inflammation and contracture, separating the soleus from the gastrocnemius was difficult, but dissection was carried out in a slow and meticulous manner. The posterior tibial vessels were identified, and a strong pulse was noted throughout. Dissection was carried down to the lower Achilles to allow for adequate rotation and positioning. Due to continued concern over the need for possible future amputation, only a portion of the soleus was rotated to cover the areas of exposed bone. The flap was tacked with a 3-0 Vicryl (Cincinnati, OH: Johnson & Johnson) which provided complete coverage.

**Figure 5:**
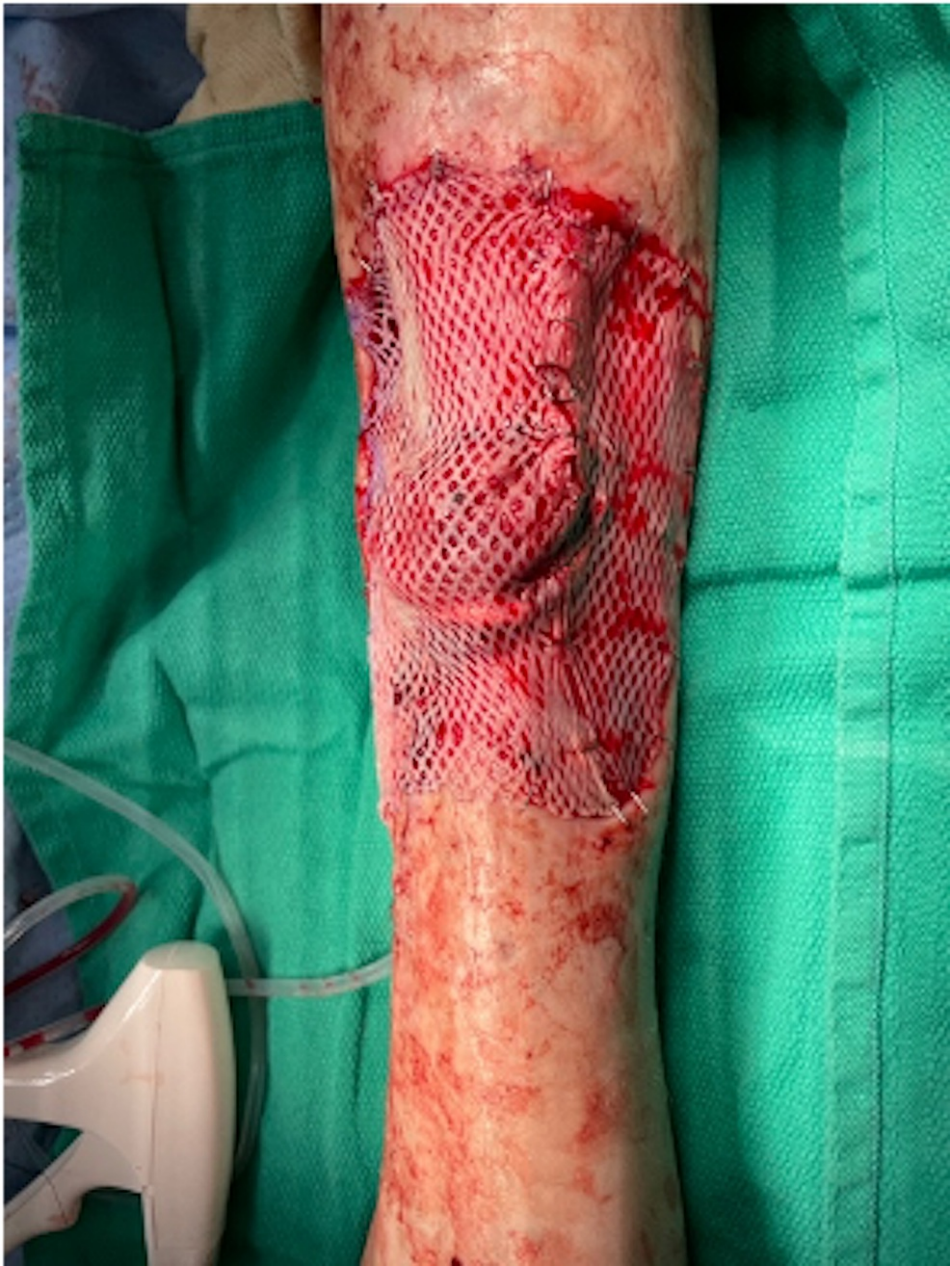
Completion soleus flap with 1:1.5 meshed split-thickness autograft. Ankle oriented toward the inferior portion of the image. Medial leg oriented toward the left side of the image.

The wound and flap were then irrigated, hemostasis was achieved, and a Jackson-Pratt (JP) drain was placed within the soleal dissection cavity. The donor site incision was closed. Skin graft was taken from the upper left thigh, meshed 1:1.5, secured in the defect with staples, and covered with Xeroform and VAC dressing (Figure 5). Forty milliliter of 0.25% Marcaine was used to obtain a fibular block and injected into the remaining gastrocnemius muscle, as well as into the donor site. The donor site was covered with Allevyn gauze. The procedure was concluded, and despite approximately 200 cc of blood loss during ligation of a soleal vein, the patient tolerated the procedure well and was stable during the course of the intraoperative and postoperative course.

The last operation planned was a VAC dressing change. This was accompanied with soleus flap drain removal and donor site dressing change. The VAC dressing was removed and the wound was found to be healthy. The drain was pulled without difficulty. The wounds were then scrubbed, including the graft, leg, and donor site. The wound was irrigated with 2 g cefazolin in 3 L normal saline solution. The donor site was dressed with Xeroform, Tegaderm and wrapped with an ACE bandage. The graft site was dressed with Xeroform before applying negative pressure wound therapy for sterile coverage.

**Figure 6:**
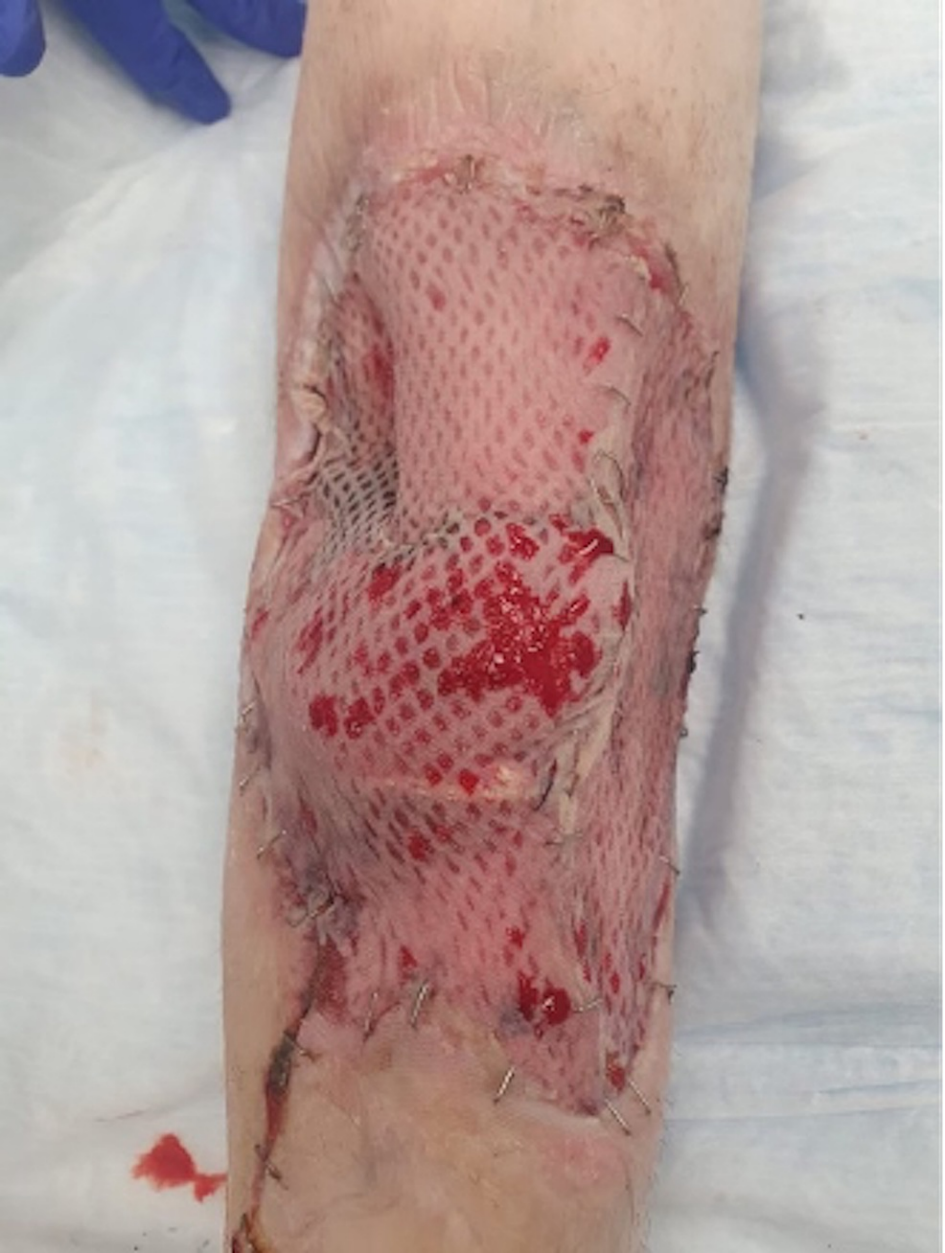
Uptake of split-thickness skin graft and following 10 days of negative pressure wound therapy. Ankle is oriented toward the inferior portion of the image. Medial leg is oriented to the left side of the image.

Approximately seven days later, the VAC was taken down again to reveal a graft that had taken up well without sign of infection and the result was considered excellent (Figure 6). In preparation for hospital discharge, the patient was seen by physical therapy/occupational therapy (PT/OT). She was able to ambulate upwards of 35 feet with a four-wheeled-walker. Subsequent disposition evaluation was for one-two weeks of sub-acute rehabilitation (SAR). The patient was subsequently discharged and successfully completed her SAR placement. After SAR discharge, she returned home, was able to abstain from further substance abuse, was able to obtain and maintain employment, and continued rehabilitation on an outpatient basis.

## Discussion

The opioid epidemic shows no signs of slowing in innercity communities or the United States at large. In Baltimore, Maryland, illicit opiate use has devastated portions of the community, resulting in some of the highest rates of HIV among drug users in the country (18% vs 9% nationally) [9,10]. And when considering that an estimated one in five intravenous drug users in Baltimore is suffering from soft tissue infection, much of the burden to care for these complex surgical patients lies on local community hospitals [11-13]. Surgical teams, when deciding how best to treat a threatened extremity, should ideally strive for limb salvage when feasible, particularly in those patients who exhibit a strong desire to abstain from illicit substance use. However, the tools and know-how may be lacking in a community-based setting which does not commonly attempt limb salvage therapy.

Previous reports have extensively documented the importance of the soleus flap for salvage of devastating lower extremity trauma. But as demographics of the United States evolve, and the use of intravenous drugs of abuse continue to rise, the need for limb salvage options for severe soft tissue loss and infection increases. As limb amputation can have devastating effects on the mobility and long-term prospects of patients (especially those of a young age), we argue that lower extremity salvage techniques should be employed whenever viable. The delayed nature of this individual’s reconstruction allowed her to successfully rehab from drug use within the hospital, as well as obtain psychiatric treatment. In the eyes of the authors, this case represents an example of limb salvage therapy with an excellent outcome as the patient was able to successfully rehab her injury, abstain from opiate use, and is able to maintain employment.

## Conclusions

The challenges treating patients within an innercity community are ever-changing. Surgeons are faced with a diverse patient population with a variety of surgical issues. The rise of the opiate epidemic within innercity America has presented a unique set of challenges as these patients can easily develop chronic, complex, limb-threatening infections. The ability to salvage these extremities, using techniques adapted from high-velocity lower extremity trauma, can and should be adopted into surgical practice when appropriate. When considering chronic limb-threatening infection to the middle or distal third lower extremity, we believe using a rotational soleus flap is a feasible, time-tested, and reliable method to provide adequate soft tissue coverage. The technique described allows patients to detox from illicit substance use as they undergo sequential debridement and wound care as they demonstrate compliance to treatment and abstinence. Our methodology allows that If the patients are able to complete this stage successfully, they can be offered an attempt at limb salvage. Patients who relapse, show a low desire for abstinence, or leave the hospital to use illicit substances should be offered traditional amputation, as pedicled reconstruction will likely ultimately fail.
